# Seeing is believing: How citizen science challenges riparian misconceptions

**DOI:** 10.12688/openreseurope.19783.1

**Published:** 2025-03-31

**Authors:** Bruna Gumiero, Leonardo Veronesi, Luisa Galgani, Francesco Di Grazia, Alessio Corsi, Riccardo Gaetano Cirrone, Steven Arthur Loiselle

**Affiliations:** 1 Center for Colloid and Surface Science, Florence, Tuscany, Italy; 2 University of Bologna, Bologna, Emilia-Romagna, Italy; 3 National Network of Biodiversity, Istituto Superiore per la Protezione e la Ricerca Ambientale, Rome, Lazio, Italy; 4 European Citizen Science Association, Berlin, Germany; 5 Department of Biotechnology, Chemistry and Pharmacy - DBCF, University of Siena, Siena, Italy; 6 National Biodiversity Future Center, Palermo, Italy; 7 Department of Social, Political and Cognitive Sciences, DISPOC, University of Siena, Siena, Italy; 8 National Research Council (CNR) Institute for Marine Sciences (ISMAR), Forte Santa Teresa, Pozzuolo di Lerici, Italy

**Keywords:** Riparian vegetation; citizen science; riparian zone management; ecosystem services; citizen engagement; public participation.

## Abstract

This open letter discusses the importance of riparian vegetation and the misconceptions surrounding its management. It highlights the essential ecosystem services provided by riparian zones, such as flood regulation, sediment control, shading, microclimate regulation, and habitat diversity. Despite these benefits, public misunderstanding often leads to harmful policies like clear-cutting and channelization. In this brief communication, we emphasize the role of citizen science in addressing public perception by involving local communities in environmental surveys, providing examples of a novel approach to monitor and manage riparian forests. This newly developed process involves volunteer training, data collection using a smartphone app, data analysis, and dissemination of results. The collected data is validated by experts and used to inform decision-making. This hands-on engagement helps replace anecdotal beliefs with evidence-based understanding, fostering better public support for conservation efforts.

## Introduction

Water courses constitute a dynamic sequence of ecosystems that exhibit both longitudinal variations, from the source to the mouth, and lateral diversity. The well-vegetated riparian corridors (Riparian zones), integral to these water courses, play a crucial role, becoming inseparable from the river itself. The components of these corridors are primarily influenced by a flooding regime characterized by high temporal and spatial variability affecting soil texture, water availability, and nutrient supply. In their natural state, this flooding regime gives rise to a mosaic landscape featuring both vegetated and bare fluvial landforms, serving as hierarchically organized habitats ( Gurnell *et al.*, 2016). Riparian zones harbor unique biotic communities, comprising species that thrive in conditions of high water and nutrient availability while also enduring shear stress and temporary submersions (hygrophilous forests) ( Del Tánago *et al.*, 2021).

Given their location and topography, floodplains are likely to form wetlands, temporary if not permanently. Even above the water table, the soil is likely to remain close to saturation because of the capillary fringe effect. The dependence of riparian zones on the flooding regime, considered along four dimensions: longitudinal (upstream–downstream), lateral (hillslope-channel), vertical (hyporheic-channel bed), and temporal, sets them apart as functionally distinct from purely terrestrial or aquatic lentic ecosystems ( Tockner *et al.*, 2000). Riparian zones are present in all biomes from tropical rainforests to arid and arctic deserts and range from large floodplain–river systems draining millions of cubic meters of water annually at a continental scale to small temporary streams.

Despite the provision of essential ecosystem services, misconceptions about riparian areas—such as the belief that they increase flooding, attract dangerous wildlife, or reduce land usability—frequently lead to policies promoting clear-cutting, channelization, or "tidying up" riverbanks. These misconceptions, reinforced by generational beliefs and selective personal experience, create resistance to new information, making it difficult for scientific findings to penetrate public consciousness and influence political decision-making. Such perceptions present a major challenge for scientists and policymakers working to implement conservation and sustainable land management strategies. Overcoming them requires not only legal protections for riparian zones but also educational efforts to shift public understanding. Effective communication, demonstrations of successful conservation projects, and engagement with local stakeholders are crucial in bridging the gap between what science tells us and what communities believe. Without addressing these misunderstandings, decision-makers may continue to favour short-term interventions that ultimately degrade both environmental and economic resilience.

This document seeks to examine the perceptions and misconceptions surrounding riparian vegetation management, as well as identifying the role that citizen science can play in bridging the gap between scientific knowledge and society through experiential learning. To illustrate this, an example of a citizen science monitoring approach is presented, followed by an overview of the diverse ecological functions of riparian systems, concluding with practical suggestions for enhancing public engagement and environmental stewardship.

## Societal perception of riparian zones

**Figure 1.**
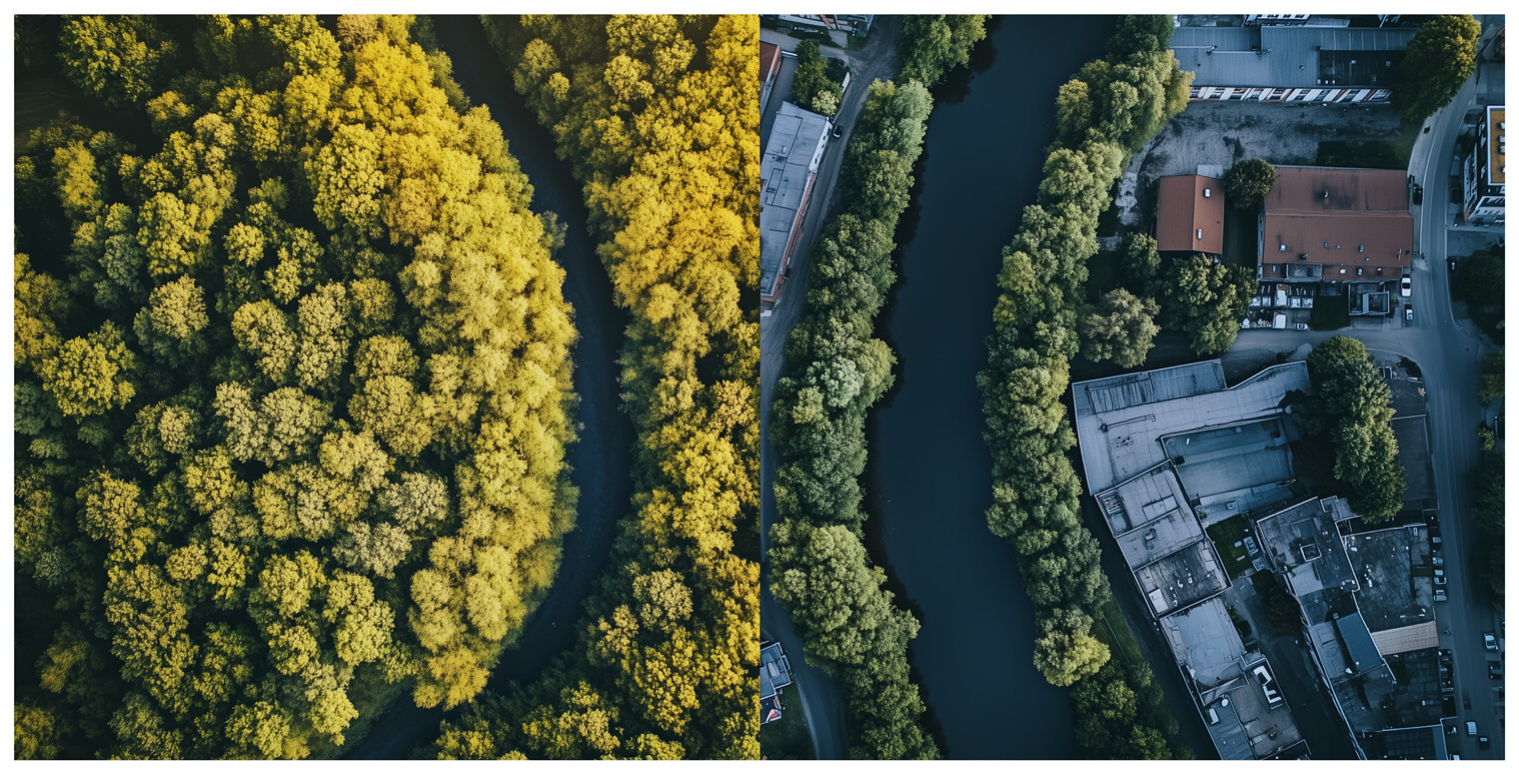
Allowing natural vegetation growth along riverbanks to occur versus streams channeled through urbanized areas.

Scientific research underscores the importance of river restoration in enhancing biodiversity, water quality, and flood resilience ( Basak *et al.*, 2021; Smith *et al.*, 2014; Vermaat *et al.*, 2016). A key principle is allowing natural processes, such as vegetation growth along riverbanks, to occur ( Feld *et al.*, 2011; Wohl *et al.*, 2005) ( Figure 1).

However, public perception often conflicts with scientific consensus. While scientists advocate for the ecological benefits of riparian vegetation, the public frequently sees it as hazardous, favoring artificial river "cleaning" practices contrary to evidence-based recommendations. For instance, lay perceptions of in-stream wood often emphasize risk and advocate its removal, despite its recognized ecological value by experts ( Chin *et al.*, 2014; Piegay *et al.*, 2005). Even after flood events, public attitudes largely favour interventions over natural processes ( Ruiz-Villanueva *et al.*, 2018).

Although increased biodiversity was shown to positively affect mood and well-being ( Cameron *et al.*, 2020), public perceptions of river ecosystems are shaped more by aesthetic and cultural values ( Thiele *et al.*, 2019). Experts sometimes fail to fully integrate these socio-cultural dimensions into restoration strategies ( Le Calvez *et al.*, 2021; Schultz *et al.*, 2022). Research suggests that socio-cultural values significantly influence public views on river management ( Garcia *et al.*, 2020). Historical practices also shape modern perceptions, though restoration efforts can gradually increase community appreciation of restored rivers ( Polednikova & Galia, 2021).

For example, a survey by Verbrugge and van den Born (2018) of a Dutch community near a planned river restoration found a positive reception, particularly regarding flood safety. Respondents also highlighted enhanced scenic beauty, social connections, and recreational value, illustrating the importance of community engagement in fostering public support.

Engaging local stakeholders can lead to better outcomes, as restoration projects often enhance appreciation for rivers’ ecological and social value ( Polednikova & Galia, 2021; Verbrugge & van den Born, 2018). Participatory practices, as emphasized by the EU Water Framework Directive, are crucial for incorporating cultural and social values into successful restoration efforts ( Schultz *et al.*, 2022).

At a European scale, narratives around river restoration remain polarized. While organizations like WWF advocate scientific approaches, political interests often resist, favouring outdated methods. Public opinion reflects similar divides, as seen in Italy, where differences between experts and the public regarding river landscapes persist ( Le Lay *et al.*, 2013). Cultural values shape these attitudes, varying across contexts ( Cottet *et al.*, 2013; Ruiz-Villanueva *et al.*, 2018). Ultimately, there is no universal solution. Successful river restoration must balance scientific evidence with local socio-cultural factors, fostering an integrated approach that benefits both biodiversity and communities.

## RiVe: a citizen science approach to riparian vegetation monitoring

The challenges posed by climate and biodiversity emergencies call for collaborative approaches to river management, prompting the question, “How should these efforts be applied?”. With the rising prominence of policies addressing flood risks and ecological restoration, river scientists are increasingly involved in co-designing and recommending solutions like rehabilitation, restoration, and rewilding. Citizen science plays a crucial role in defining scenarios and fostering adaptive management, bridging natural and social sciences in designing sustainable strategies.

By involving local communities in the collection and interpretation of environmental data, citizen science transforms abstract scientific concepts into tangible, firsthand experiences. For example, when residents actively monitor riparian vegetation, water quality, or biodiversity, they begin to see the direct benefits of these ecosystems—such as improved water clarity, reduced erosion, and thriving wildlife. This hands-on engagement helps dispel misconceptions, replacing anecdotal beliefs with evidence-based understanding.

Well-designed citizen science projects for riparian zones should focus on raising community awareness about the ecological importance of riparian forests and their role in providing essential ecosystem services. Educating citizens fosters a deeper connection between people and their local river environments. In addition to awareness-building, citizen science initiatives should contribute new knowledge by engaging citizens in monitoring riparian forest quality using simple, scientifically valid methods. These methods must be both effective and accessible to non-experts, ensuring inclusivity.

Data collected during citizen science activities should be stored in a shared, open-access database, validated by experts, and made publicly available. Participants should actively engage in data analysis, incorporating their local knowledge to complement expert insights. Sharing results with local authorities and the broader community is essential for promoting actionable outcomes.

Projects like OTTERS ( https://otters-eu.aua.am/) or the European framework like Mission Ocean and Water 2030 aim to standardize methodologies across Europe, enabling comparison and aggregation of results on a continental scale. This approach not only enhances scientific robustness but also ensures the scalability and impact of citizen science in addressing riparian zone management challenges.

**Figure 2.**
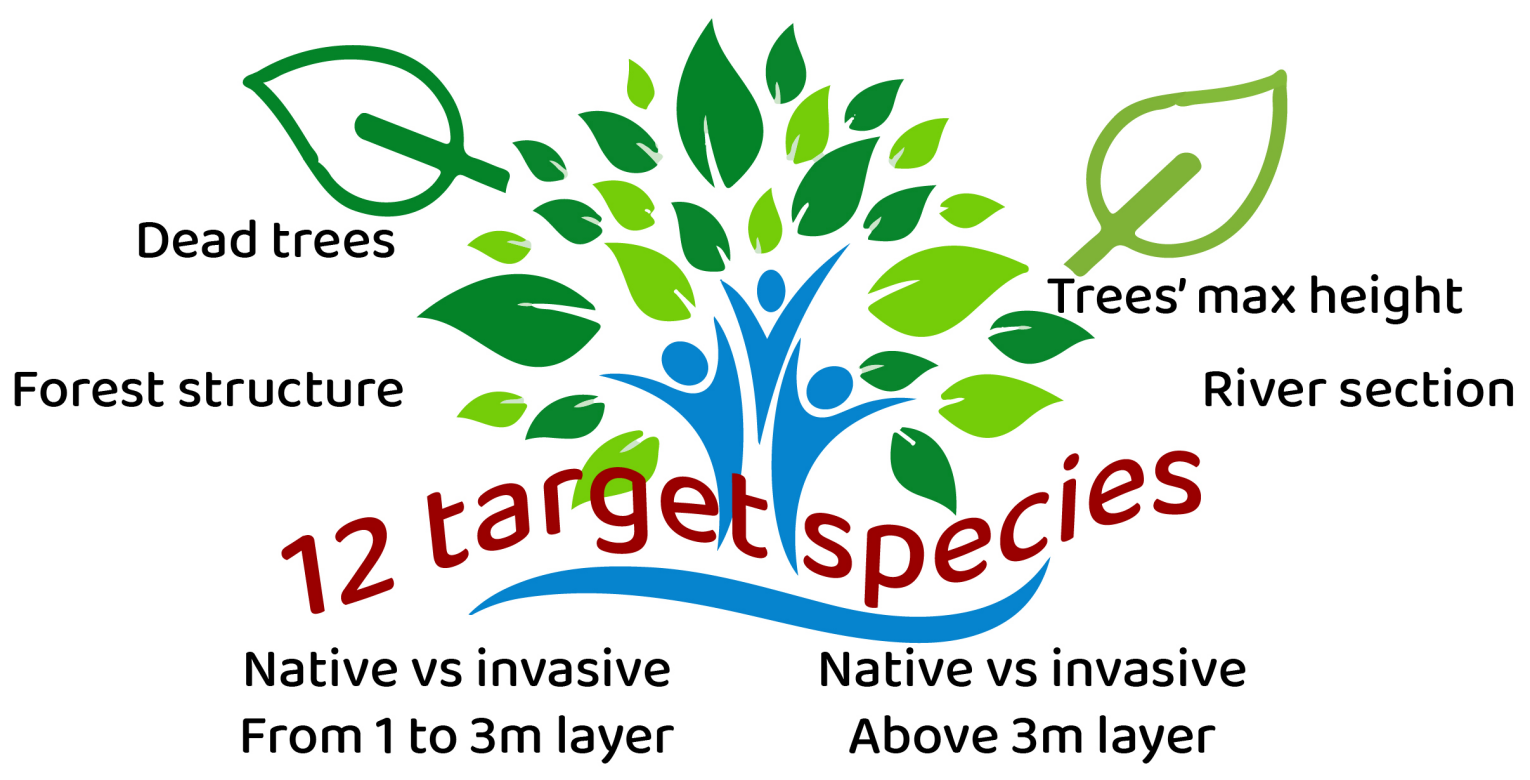
The RiVe project structure.

While several large-scale initiatives exist to monitor water quality, hydrology, geomorphology, and biodiversity, no dedicated citizen science project has focused specifically on riparian forests—until now. To address this gap, the RiVe methodology was developed in Italy as a novel approach for monitoring the quality of riparian forests through the systematic collection of targeted data. RiVe serves as a Citizen Science tool, complementing other large-scale monitoring methods for riparian zones, such as remote sensing surveys like the QBR-GIS introduced by Segura-Méndez *et al.* (2023). Developed in Italy in 2020 by the National Biodiversity Network – ISPRA and the Citizen Science Observatory ( Gumiero *et al.*, 2024) ( https://www.nnb.isprambiente.it/vegetazioneriparia/inviasegnalazioni-en.html RiVe is designed around four key phases: volunteer training, data collection, data analysis and dissemination of results to decision makers and society ( Figure 2). Data collection is facilitated by an open-source smartphone app (ODK). During fieldwork, volunteers define a sampling area of approximately 10×15 meters and complete a detailed form assessing forest structure. They also record the presence and abundance of 12 target woody species, distributed across two layers, which are selected based on their ecological significance and their ability to reflect the hydrological conditions and disturbance levels of the area. The collected data is subject to validation by project coordinators, ensuring scientific rigor. Furthermore, the collected data can be summarized in two indices, one is the invasiveness index (iRI) and the other is the riparian index (iRiVe) which estimates the overall quality of the riparian forest.

## Riparian vegetation: functions and ecosystem services

**Figure 3.**
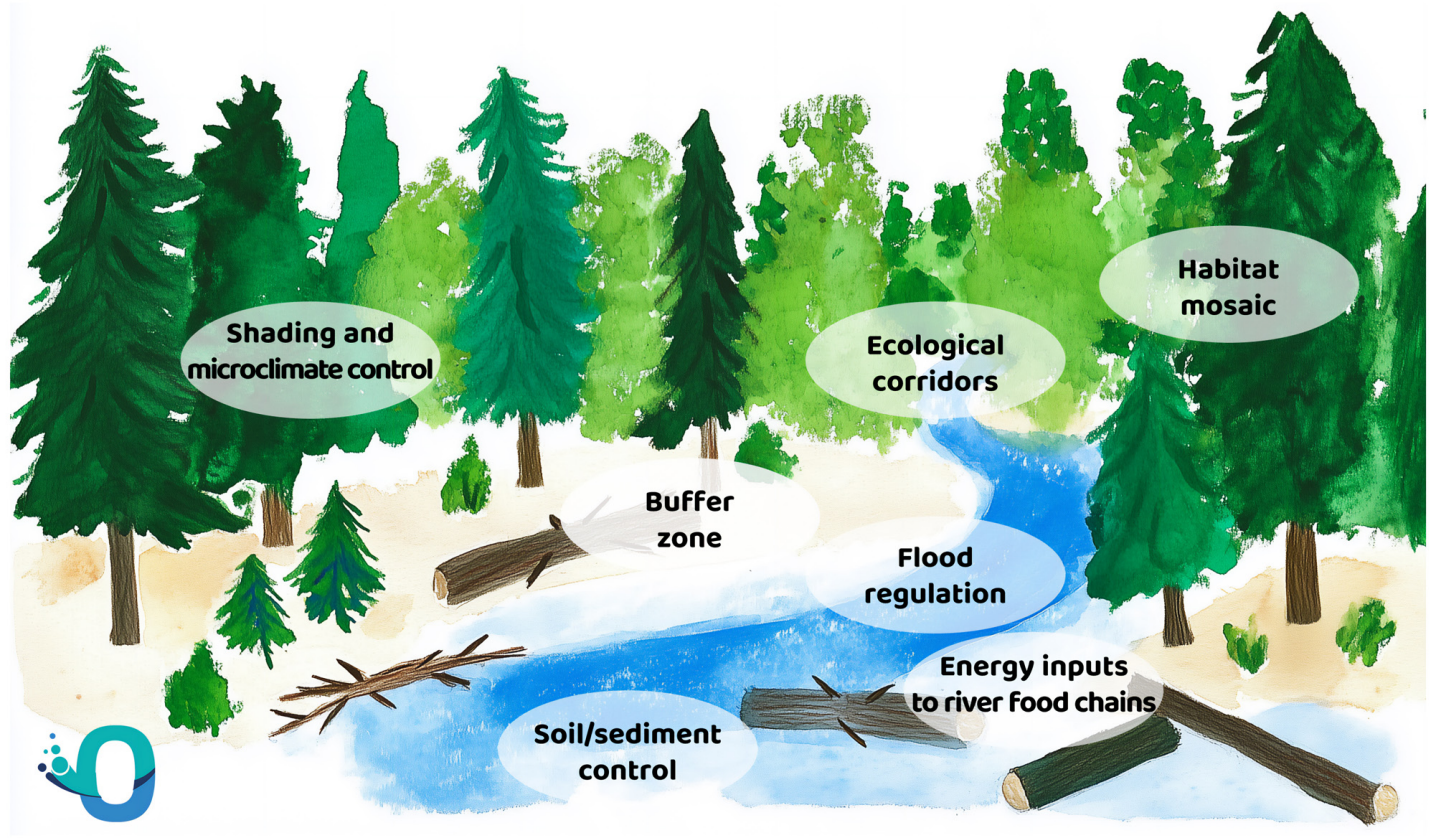
Functions of riparian vegetation in maintaining healthy rivers and ecosystems.

Riparian vegetation has many key functions to maintain rivers in a healthy state. As an example, it helps improve soil texture and provides mechanical stabilization through its root system, increasing the content of organic matter and nutrient retention. The ecological functions of riparian vegetation are many: ( Figure 3).

- **Flood Regulation.** Riparian vegetation, composed of trees, shrubs, and grasses adapted to wet areas, mitigates floods by slowing floodwater, allowing groundwater recharge, and reducing downstream flood peaks during heavy rains ( Rak *et al.*, 2016). Vegetation adds roughness to water flow, reducing speed and energy, which facilitates infiltration and replenishes aquifers ( Keesstra *et al.*, 2018; Riis *et al.*, 2020). Additionally, riparian areas provide a steady release of water during dry periods, benefiting stream health and irrigation.
- **Sediment Control.** Dense root systems of riparian vegetation stabilize soils and control sediment movement. Species native to riparian zones are particularly effective in limiting erosion compared to occasional species ( Feld *et al.*, 2018; Simon & Collison, 2002). Mixed vegetation, including woody and herbaceous plants, reduces soil runoff, with vegetated soils showing significantly less erosion than bare soils ( Beeson & Doyle, 1995). Large Woody Debris (LWD) also shapes riverbeds, influences sediment deposition, and enhances channel stability ( Swanson *et al.*, 2021).
- **Shading and Microclimate Regulation**. Riparian vegetation moderates’ microclimates by reducing wind speed, evaporation, and excessive light penetration. Canopy shading prevents algal overgrowth, lowers water temperatures during heatwaves, and supports oxygen solubility, which benefits aquatic biodiversity ( Garner *et al.*, 2017; Trimmel *et al.*, 2018). Shading contributes to a diverse habitat for fish and macroinvertebrates, fostering greater riverine biodiversity ( Rios & Bailey, 2006).
- **Buffer Zone.** Riparian zones filter sediments, nutrients, and pollutants, improving water quality through physical and biological processes ( Gumiero *et al.*, 2013; Gumiero *et al.*, 2015; Pinay *et al.*, 2000). They effectively remove dissolved inorganic nitrogen and phosphorus present in excess in water runoff from surrounding agricultural or urban areas, which can otherwise lead to eutrophication. One of the main biological processes capable of removing nitrate by reducing it to atmospheric nitrogen (N _2_) is denitrification, which occurs in riparian micro-sites in anaerobic conditions rich in organic carbon ( Gumiero *et al.*, 2011). In this case, riparian vegetation has an indirect but fundamental role in providing food to the denitrifying bacterial community. The assimilative absorption by plants and microorganisms also contributes significantly to the removal, even if temporary, of inorganic nitrogen and phosphorus. Riparian vegetation also degrades pesticides and contaminants through metabolic and adsorption processes ( Aguiar *et al.*, 2015).
- **Energy Inputs to Food Chains.** Riparian vegetation supplies Coarse Particulate Organic Matter (CPOM) like leaves and woody debris, forming a food source for aquatic organisms. CPOM sustains detritivores and decomposers, with some aquatic species timing their life cycles to peak CPOM availability ( Deegan & Ganf, 2008; Giri & Laub, 2025).
- **Habitat Diversity and Channel Morphology.** Riparian vegetation enhances habitat heterogeneity along riverbanks, creating refuges and nursery areas for aquatic and terrestrial species. Large woody debris forms essential habitats, such as pools that sustain fish during extreme conditions like floods and droughts ( Tabacchi *et al.*, 2003). Riparian zones support diverse flora and fauna, including amphibians, mammals, birds, and fish, with many species depending on these areas during their life cycles ( Bateman & Merritt, 2020; Naiman *et al.*, 1993).
- **Ecological Corridors.** Riparian zones serve as natural ecological corridors, facilitating species movement and connecting habitats. They ensure longitudinal, lateral, and vertical connectivity within riverine ecosystems, supporting biodiversity and energy flow ( Naiman & Décamps, 1997). These corridors play a crucial role in reducing habitat fragmentation and genetic isolation, promoting ecological resilience ( Camporeale *et al.*, 2013). The EU’s 2030 biodiversity strategy ( European Commission, 2021) emphasizes riparian corridors as part of a coherent and resilient trans-European nature network.

These distinctive characteristics, coupled with their intermediary position between aquatic and terrestrial environments, empower riparian zones to fulfill essential functions and provide "ecosystem services" crucial to society. Ecosystem functions refer to natural processes and interactions within an ecosystem. These functions occur regardless of human use while ecosystem services are the benefits that humans derive from these functions. In other words, ecosystem services translate ecological processes into direct human value. Functions are what nature does, while services are how those functions benefit us.

**Figure 4.**
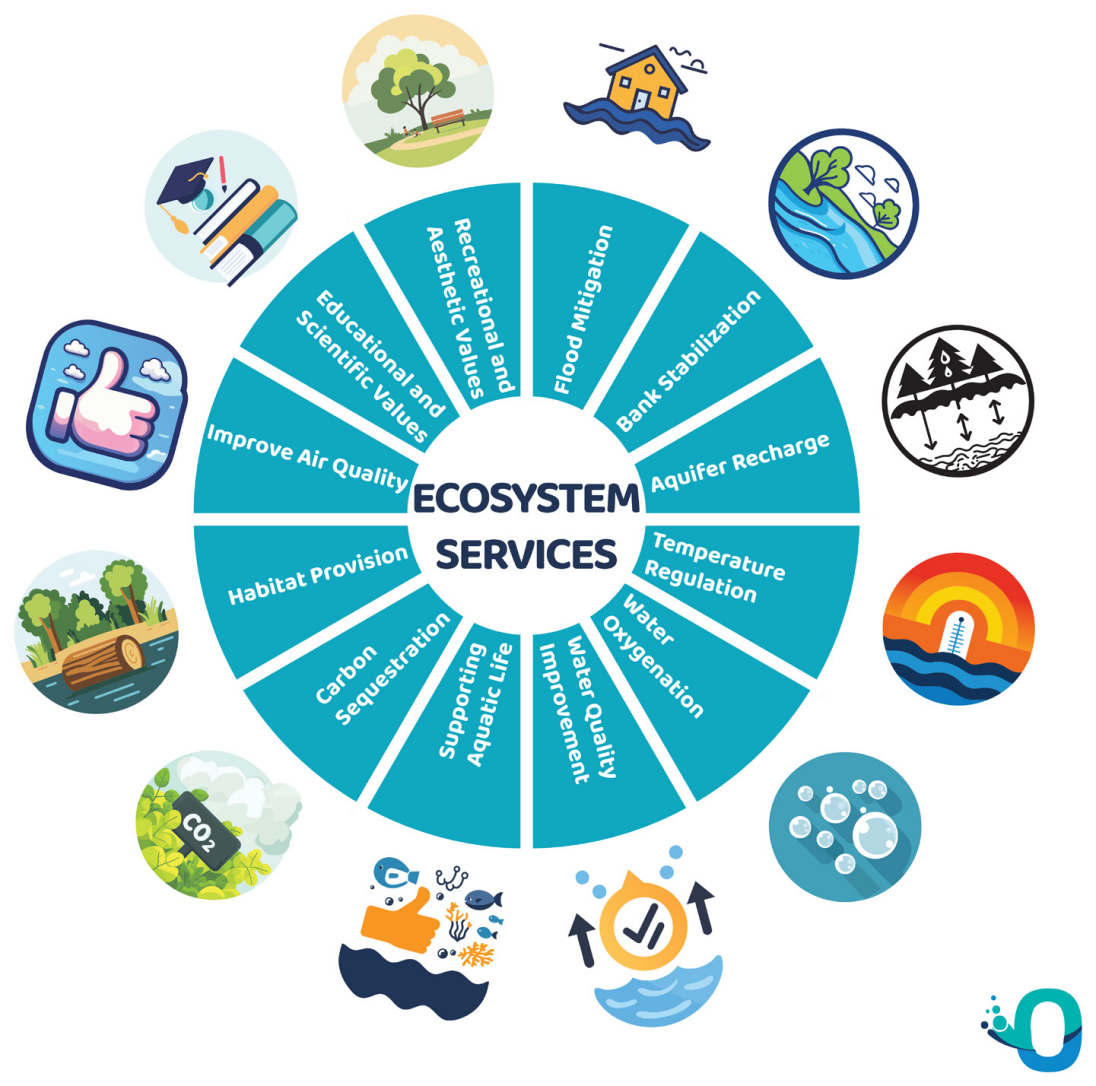
Ecosystem services of riparian vegetation.

Riparian vegetation offers multiple ecosystem services ( Figure 4):

- **Flood Mitigation:** Riparian vegetation helps mitigate flooding by slowing down and absorbing floodwater, reducing the impact of extreme events on both riverbanks and adjacent areas.
- **Bank Stabilization**: The root systems of riparian plants help stabilize riverbanks, reducing erosion and maintaining the integrity of the river channel.
- **Aquifer Recharge**: The vegetation in riparian zones facilitates the recharge of shallow aquifers by allowing water to infiltrate the soil, contributing to groundwater storage essential for sustaining river flow during dry periods.
- **Temperature Regulation**: The shading effect of riparian vegetation helps regulate water temperature in rivers, preventing excessive heating. This is particularly important for maintaining suitable conditions for aquatic organisms and preventing the proliferation of certain unwanted species.
- **Water Oxygenation**: By shading and cooling water, riparian vegetation contributes to higher dissolved oxygen levels, creating a healthier environment for aquatic life.
- **Water Quality Improvement**: Riparian ecosystems act as natural filters, reducing sediment and nutrient levels in water through the absorption and retention of pollutants, thus enhancing water quality downstream.
- **Supporting Aquatic life**: Leaves and woody material from riparian vegetation serve as a crucial source of food, especially Coarse Particulate Organic Matter (CPOM), supporting the energy needs of aquatic organisms.
- **Habitat Provision**: Riparian areas offer diverse habitats for various species of flora and fauna, supporting biodiversity. The complexity of these ecosystems provides niches and breeding grounds for numerous organisms.
- **Carbon Sequestration**: Riparian vegetation plays a role in carbon sequestration, helping to mitigate climate change by storing carbon in plant biomass and soil.
- **Improve air quality**: The riparian forest, like all woods and forests, improve air quality by reducing CO _2_ levels.
- **Educational and Scientific Value**: Riparian zones offer opportunities for environmental education and scientific research, contributing to our understanding of ecosystems, biodiversity, and hydrological processes.
- **Recreational and Aesthetic Values**: Riparian zones provide opportunities for recreational activities such as fishing, canoeing, hiking, and birdwatching. The aesthetic appeal of these areas contributes to human well-being and cultural value.

## Challenges in riparian zone management: addressing policy and perception barriers

Riparian zones play a central role in maintaining ecological balance and supporting human well-being, delivering essential ecosystem services. However, their sustainable management is hampered by ecosystem complexity, spatial and temporal dynamics, and increasing pressures from urbanization, agriculture, deforestation, river engineering, and invasive species ( Zipperer *et al.*, 2020). These stressors threaten biodiversity and diminish the capacity of riparian zones to provide critical services.

Degradation of riparian vegetation and floodplains exacerbates flood risks, leading to significant economic and ecological consequences ( Hallegatte *et al.*, 2013; Poff *et al.*, 1997). Despite these challenges, policymakers often underestimate the indirect costs of reduced ecosystem services, underscoring the urgent need for broader recognition of riparian functions. In water-scarce regions, competition for water resources—driven primarily by agriculture and urban development—further stresses these biodiversity hotspots ( Singh *et al.*, 2021; Urbanič *et al.*, 2022).

In Europe, riparian zones are insufficiently addressed by pivotal policies like the EU Water Framework Directive (WFD) ( European Union, 2000). Fragmented and occasionally conflicting directives, such as the WFD, Floods Directive, and Renewable Energy Directive, impede integrated management efforts ( Gumiero *et al.*, 2013). For instance, defining riparian zones by fixed widths fails to capture their ecological complexity, hindering sustainable management. Emphasizing river basin management based on the "designing with nature" philosophy can better balance ecological and social considerations, reducing the reliance on heavy interventions such as river "cleaning" ( Lilli *et al.*, 2020).

A coordinated policy approach is essential, integrating conservation, agriculture, and water management while acknowledging riparian zones as interconnected with broader ecosystems. Policymakers should adopt adaptive, socio-economic strategies emphasizing collaboration, stakeholder engagement, and ongoing monitoring. Bridging the gap between science and policy through co-creation models and citizen science can enhance decision-making and promote integrated management.

Global initiatives such as the EU Green Deal and the Nature Restoration Law (NRL) ( Regulation 2024/1991) offer opportunities to improve riparian zone management. However, the success of the NRL depends on Member States' commitment to restoring rivers and riparian corridors, crucial for sustaining freshwater biodiversity and ecosystem services ( Albert *et al.*, 2021). A lack of political will and funding remains a major obstacle, with freshwater conservation investments lagging significantly behind those for terrestrial and marine ecosystems ( Maasri *et al.*, 2022).

Effective implementation of the NRL and preserving natural rivers are imperative for maintaining the balance of freshwater ecosystems. Immediate action is needed to protect these environments and ensure long-term benefits for biodiversity and human well-being ( Stoffers *et al.*, 2024).

Despite the ecological importance of riparian ecosystems, progress in improving their condition across Europe has been limited due to fragmented knowledge, poor communication among stakeholders, and insufficient tools for assessment and coordinated action. The COST Action CONVERGES focused on riparian ecosystems, aiming to create a European network that unites diverse knowledge on riparian vegetation across physical, ecological, and societal domains. It seeks to synthesize knowledge, improve stakeholder communication, identify gaps, and develop effective management tools to enhance ecosystem restoration and policy alignment ( https://converges.eu/converges/). When public opinion is dominated by outdated or incorrect understandings of environmental realities, political leaders may hesitate—or outright refuse—to act on scientific recommendations, fearing electoral backlash or economic disruption. The result is often a dangerous delay in adopting policies that could mitigate risks, improve sustainability, and ensure long-term resilience.

Overcoming these barriers requires persistent public education, transparent communication of scientific data, and policies that integrate both expert knowledge and local engagement. Without addressing these deep-seated misperceptions, the gap between what is scientifically known and what is politically acted upon will continue to hinder necessary adaptation and progress.

## Conclusions – the way forward

Citizen science plays a pivotal role in enhancing communication between researchers, policymakers, and the public. By involving non-experts in data collection, it makes scientific information more accessible and relevant, facilitating its integration into decision-making processes. This engagement also fosters trust in scientific findings, as individuals witness the results emerging from their own environments, rather than perceiving science as something distant or imposed. Moreover, citizen science cultivates a sense of ownership and responsibility. When people actively contribute to data collection, they are more likely to support conservation efforts that are based on their own observations. For example, when local experiences—such as increased flooding after the removal of riparian vegetation—align with scientific predictions, community members become powerful advocates for evidence-based policies.

In this context, the OTTERS Project emphasizes the essential role of citizen science in improving communication among researchers, policymakers, and the public, while advocating for greater recognition of this participatory process. The project seeks to develop scientifically valid, standardized methods to create a European network for shared and comparable data, further strengthening the integration of citizen science into decision-making. To achieve these objectives, OTTERS promotes the RiVe method, currently the only method in Europe that focuses exclusively on assessing the quality of riparian forests using a semi-quantitative approach. The method is being refined to ensure its applicability at a European scale and to meet the evolving challenges identified. Additionally, the integration of this method with remote sensing techniques is being explored. Since field analysis often complements sophisticated remote sensing methods, the synergy between these two approaches is expected to yield more accurate, comprehensive results.

In conclusion, citizen science serves not only as an educational tool but also as a catalyst for social change. By transforming passive observers into active participants, it reduces resistance to scientific recommendations, fosters a culture of environmental stewardship, and ultimately leads to better-informed policies and more sustainable management of natural resources.

### Disclaimer

*The views expressed in this article are those of the authors. Publication in OPEN RESEARCH EUROPE does not imply endorsement by the European Commission’s Horizon Europe funding programme*.

## Ethics and consent

Ethics and consent were not required.

## Data Availability

No data are associated with this article.
