# Citizen-engaged research for improved perceptions of riparian systems

**DOI:** 10.12688/openreseurope.19783.2

**Published:** 2025-10-17

**Authors:** Bruna Gumiero, Leonardo Veronesi, Luisa Galgani, Francesco Di Grazia, Alessio Corsi, Riccardo Gaetano Cirrone, Steven Arthur Loiselle

**Affiliations:** 1 Center for Colloid and Surface Science, Florence, Tuscany, Italy; 2 University of Bologna, Bologna, Emilia-Romagna, Italy; 3 National Network of Biodiversity, Istituto Superiore per la Protezione e la Ricerca Ambientale, Rome, Lazio, Italy; 4 European Citizen Science Association, Berlin, Germany; 5 Department of Biotechnology, Chemistry and Pharmacy - DBCF, University of Siena, Siena, Italy; 6 National Biodiversity Future Center, Palermo, Italy; 7 Department of Social, Political and Cognitive Sciences, DISPOC, University of Siena, Siena, Italy; 8 National Research Council (CNR) Institute for Marine Sciences (ISMAR), Forte Santa Teresa, Pozzuolo di Lerici, Italy

**Keywords:** Riparian vegetation; citizen science; riparian zone management; ecosystem services; citizen engagement; public participation.

## Abstract

Riparian zones are vital ecological corridors that provide flood regulation, water purification, habitat diversity, and carbon storage. Yet persistent biases—such as the belief that riparian vegetation causes flooding—continue to shape policy and public opinion. Rooted in historical practices of dredging and river “cleaning,” and reinforced by political and media narratives, these biases obscure scientific evidence and hinder sustainable management. The Emilia-Romagna floods (2023–2024) illustrate how riparian forests are often scapegoated, while the true drivers of risk—urban encroachment, land-use change, and climate intensification—remain overlooked. This open letter calls for a transition from short-term interventions toward long-term resilience strategies that restore floodplains and give rivers space. We highlight the role of citizen science, through projects such as OTTERS and the RiVe method, in bridging science, policy, and society. By involving communities in monitoring and decision-making, citizen science fosters trust, enhances evidence-based policies, and promotes ecological restoration.

## Introduction

Human perceptions of landscapes are often shaped more by tradition, anecdote, and cultural narratives than by scientific knowledge. Many people hold deep-seated views about their environment—its climate, natural hazards, resources, and ecological balance—that persist despite evidence to the contrary ( Buijs *et al.*, 2011; Kahan, 2015). These misunderstandings, reinforced by generational beliefs and selective personal experiences, create resistance to new information and hinder the uptake of scientific insights in both public consciousness and political decision-making ( Leiserowitz *et al.*, 2021). Within this context, it is important to distinguish between misconceptions and biases. Misconceptions are factually incorrect ideas, whereas biases are ingrained cognitive shortcuts that perpetuate those misconceptions and shape judgments in systematic ways ( Kahneman, 2011). Together, they contribute to policy inertia: even well-documented realities—such as the role of floodplains in buffering extreme events or the benefits of riparian biodiversity—often struggle to gain political recognition, despite strong scientific consensus ( IPCC, 2022; Oreskes & Conway, 2010).

Cultural biases against science often influence politicians more strongly than scientists, since political actors operate within systems driven by public opinion, ideology, and electoral pressures ( Jasanoff, 2010). While scientists are trained to evaluate knowledge through peer review and empirical testing, politicians must balance evidence with the values, beliefs, and norms of their constituents. In societies where scepticism toward science—on issues such as climate change, vaccination, or biotechnology—is closely tied to cultural identity or political ideology, elected officials may downplay or reject scientific consensus to preserve legitimacy and support ( Gauchat, 2012; Hornsey *et al.*, 2018). 

This letter examines the biases surrounding riparian vegetation, despite their crucial function and services, and highlights the role of citizen science in bridging the gap between scientific knowledge and public perception. A citizen-science monitoring approach is presented as a case study, followed by practical suggestions for strengthening stakeholder engagement.

## Riparian forest: a fundamental component of watercourses

Watercourses represent dynamic sequences of ecosystems that vary longitudinally, from source to mouth, and laterally across their floodplains. Riparian corridors—well-vegetated transition zones along streams and rivers—are integral components of these systems and function inseparably from the watercourse itself. Their structure and dynamics are strongly shaped by flooding regimes characterized by high temporal and spatial variability, which influence soil texture, water availability, and nutrient cycling. Under natural conditions, such regimes create a shifting mosaic of vegetated and unvegetated fluvial landforms that serve as hierarchically organized habitats ( Gurnell *et al.*, 2016). Riparian zones host distinctive biotic communities, including species adapted to high water and nutrient availability, as well as to disturbances such as shear stress and periodic submersion (hygrophilous forests ( Del Tánago *et al.*, 2021). They occur in all biomes—from tropical rainforests to arid and arctic deserts—and across all scales, from vast continental river–floodplain systems draining millions of cubic meters annually to small, ephemeral streams ( Tockner *et al.*, 2000).

**Figure 1.**
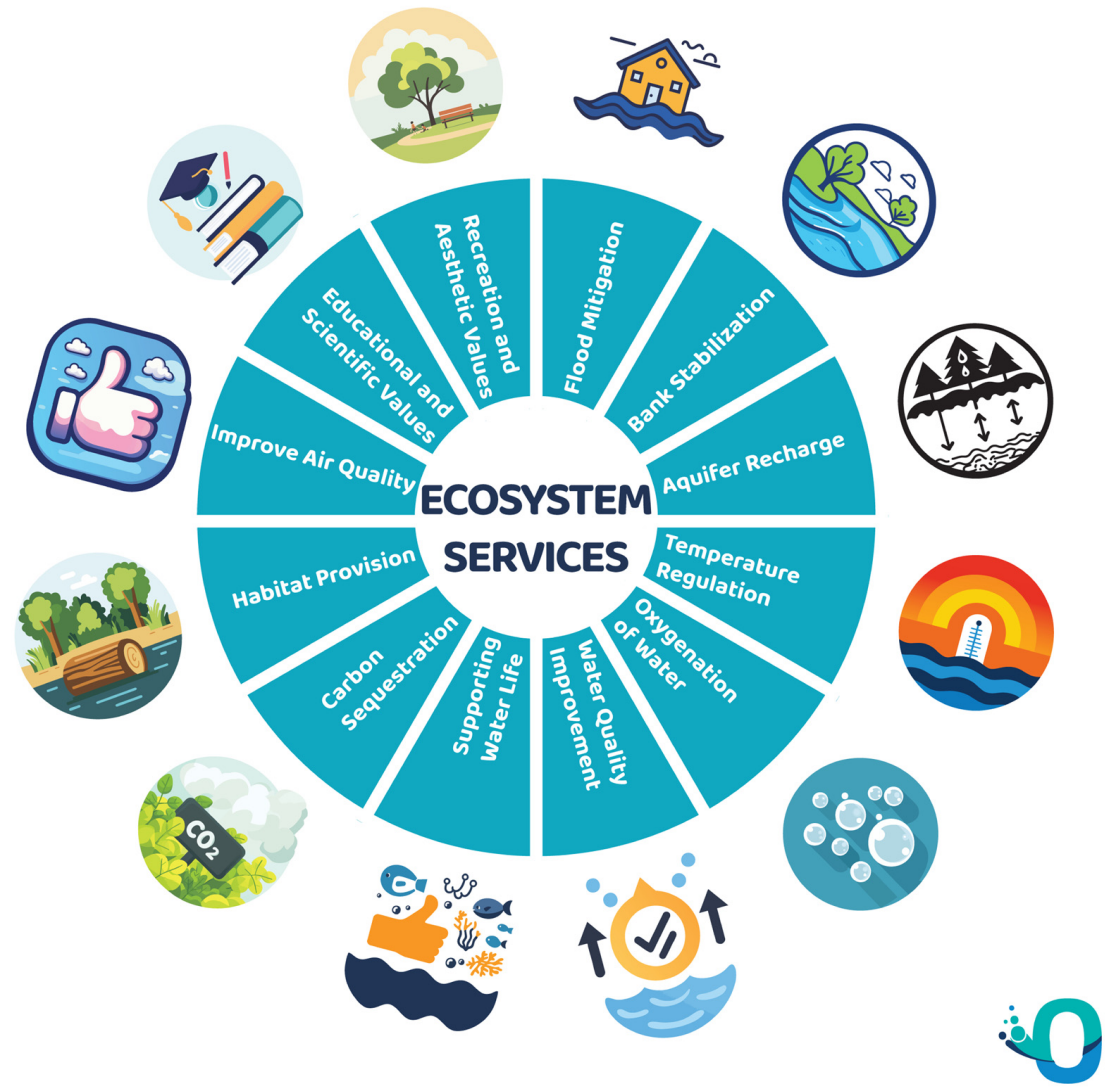
Main Ecosystem Services of Riparian Forest.

The unique ecological position of riparian zones, bridging aquatic and terrestrial environments, underpins a wide array of functions and ecosystem services vital to both ecosystems and society ( Basak *et al.*, 2021; Cole *et al.*, 2020; Smith *et al.*, 2014; Vermaat *et al.*, 2016). **Ecosystem functions** refer to natural processes and interactions, while **ecosystem services** denote the benefits humans derive from them. In essence, functions are what ecosystems *do*, whereas services are how those functions *benefit people* ( Figure 1).

Key functions and services provided by riparian vegetation include:

- **Flood regulation and mitigation.** composed of trees, shrubs, and grasses adapted to inundation—enhances surface roughness, which slows water flow and dissipates energy. This reduction in velocity promotes infiltration and aquifer recharge ( Keesstra *et al.*, 2018; Riis *et al.*, 2020), while also lowering downstream flood peaks ( Rak *et al.*, 2016). During dry periods, riparian zones act as natural reservoirs, gradually release stored water, to sustain streamflow and support irrigation.
- **Sediment control and bank stabilization.** Dense root systems stabilize soils and limit erosion, with native riparian species proving especially effective compared to occasional colonizers ( Feld *et al.*, 2018; Simon & Collison, 2002). Mixed woody–herbaceous vegetation markedly reduces soil runoff, while large woody debris (LWD) influences sediment deposition, riverbed morphology, and channel stability ( Dunn *et al.*, 2022; Swanson *et al.*, 2021).
- **Shading and microclimate Regulation**. Riparian canopies reduce wind speed, evapotranspiration, and excessive light penetration. Shading moderates stream temperature, preventing thermal stress and algal blooms, while cooler waters maintain higher dissolved oxygen levels essential for aquatic life ( Garner *et al.*, 2017; Trimmel *et al.*, 2018). Such microclimatic regulation fosters diverse fish and macroinvertebrate communities ( Rios & Bailey, 2006)
- **Water quality improvement.** Acting as buffer zones, riparian areas filter sediments, nutrients, and pollutants via physical retention and biological processes ( Gumiero *et al.*, 2013; Gumiero *et al.*, 2015; Pinay *et al.*, 2000). They play a central role in nitrogen removal through denitrification, a microbial process that converts nitrate into atmospheric N _2_ under anaerobic, carbon-rich conditions ( Gumiero *et al.*, 2011). Riparian vegetation supports this process by supplying organic matter to microbial communities. Additional pathways, such as plant uptake and microbial assimilation, further reduce nitrogen and phosphorus, albeit temporarily. Riparian zones also degrade pesticides and contaminants through metabolic and adsorption processes ( Aguiar *et al.*, 2015).
- **Energy Inputs to food webs**. Riparian vegetation contributes coarse particulate organic matter (CPOM)—such as leaves and woody debris—that serves as a primary energy source for aquatic detritivores and decomposers. Many aquatic organisms synchronize their life cycles with seasonal CPOM availability ( Deegan & Ganf, 2008; Giri & Laub, 2025).
- **Habitat diversity and channel morphology**. By shaping bank and channel structures, riparian vegetation enhances habitat heterogeneity and provides refuges and nursery areas for numerous aquatic and terrestrial species ( Bateman & Merritt, 2020). Large woody debris creates pools and shelter that are critical during floods and droughts ( Tabacchi *et al.*, 2003).
- **Ecological connectivity**. Functioning as ecological corridors, riparian zones facilitate species movement and ensure longitudinal, lateral, and vertical connectivity in riverine ecosystems ( Naiman & Décamps, 1997). This connectivity reduces habitat fragmentation, enhances genetic exchange, and strengthens ecological resilience ( Camporeale *et al.*, 2013). The EU Biodiversity Strategy for 2030 highlights riparian corridors as key elements in a trans-European nature network ( European Commission, 2021).
- **Carbon Sequestration and ait quality improvement**. Like other forests, riparian vegetation contributes to climate change mitigation by reducing atmospheric CO _2_ and sequestering carbon in biomass and soils.
- **Educational and scientific value**. Riparian ecosystems provide opportunities for ecological research and environmental education, advancing knowledge of biodiversity and hydrological processes.
- **Recreational and cultural service**. These landscapes offer recreational activities such as fishing, canoeing, hiking, and birdwatching. Their aesthetic appeal enhances human well-being and cultural identity ( Cameron *et al.*, 2020; Thiele *et al.*, 2019).

## Societal perception of riparian zones and related biases

**Figure 2.**
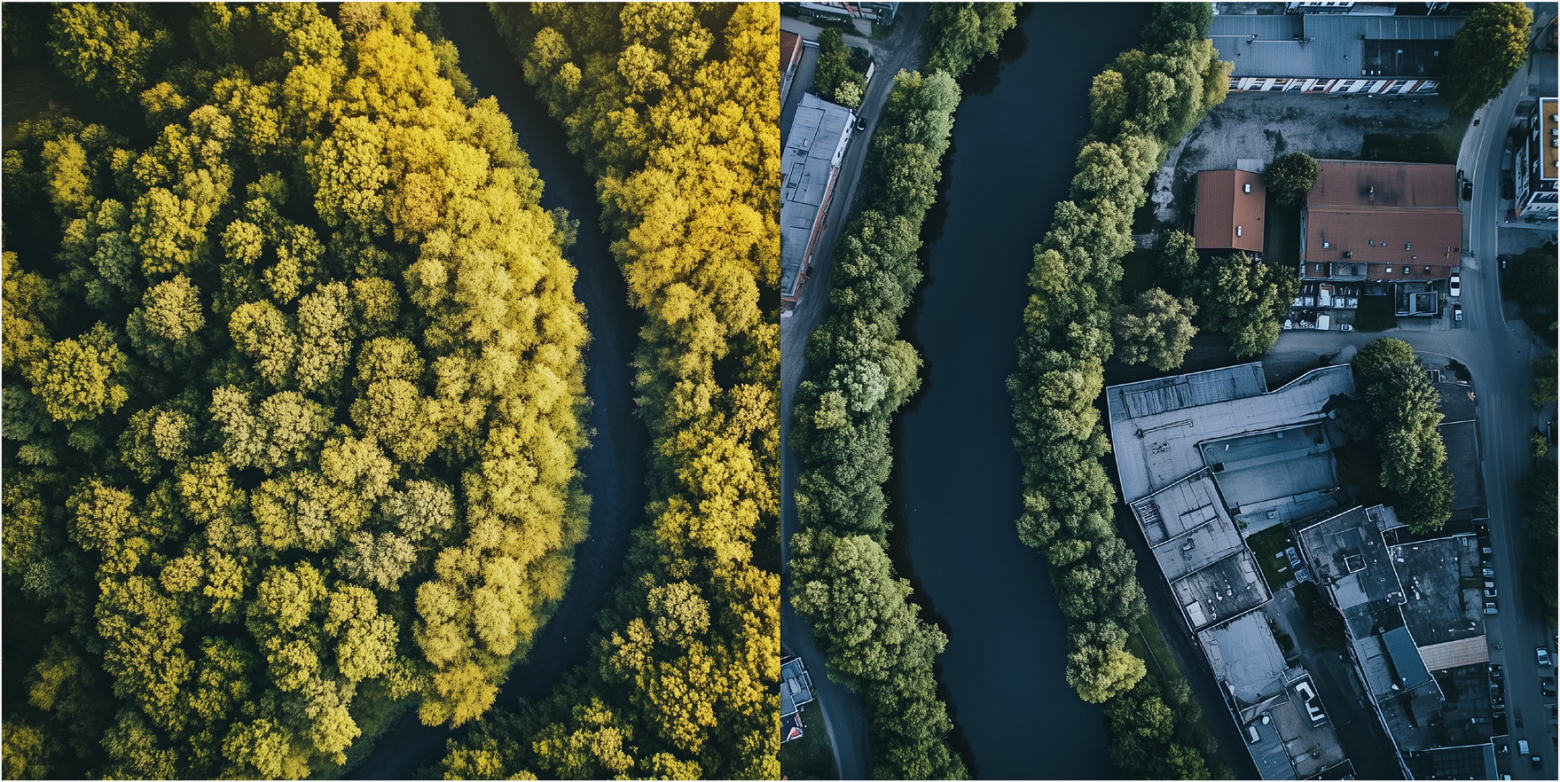
Contrasting landscapes along a river: on the left, dense riparian forests buffer the waterway; on the right, urban development closely borders the river, reducing the extent of natural vegetation.

Despite the well-documented ecosystem services provided by riparian zones, persistent misconceptions continue to shape public opinion and policy. Common beliefs include that riparian vegetation causes flooding, harbors dangerous wildlife, or reduces usable land. These perceptions have long justified interventions such as clear-cutting, channelization, and the “tidying up” of riverbanks, reinforcing the cultural expectation that “clean” rivers are safer ( Figure 2).

The Emilia-Romagna floods of 2023–2024 illustrate this gap between perception and science. While experts identified rainfall intensity, land use, and climate change as key drivers, local leaders and media attributed the disaster to a “lack of river cleaning.” Environmental groups advocating restoration were even accused of threatening public safety. Such rhetoric misrepresents ecological principles and obstructs evidence-based management. The real issue lies in human occupation of natural floodplains. In Emilia-Romagna, levees, roads, and housing confined flows, making vegetation appear more hazardous than it is. Most large woody debris threatening infrastructure, like bridges, originates from upstream landslides or unstable banks, not intact riparian forests. Well-structured forests stabilize riverbanks and intercept debris before it reaches vulnerable sites.

Blaming vegetation is politically appealing: it is visible, tangible, and inexpensive to remove. Mature forests can even provide biomass for Renewable Energy companies with valuable wood, turning clearance into an economic opportunity. Yet research shows that well-managed riparian zones mitigate, rather than exacerbate, flood risks. Restoration—not wholesale vegetation removal—is the sustainable pathway. Expanding floodplains, reconnecting rivers to natural corridors, and restoring space for water to spread are proven strategies to reduce flood peaks while preserving ecological integrity. Selective interventions may be justified in urban contexts, but large-scale clearance undermines resilience. Even in dammed or channelized rivers, riparian trees can be partly maintained if the space between banks allows.

Socio-cultural values strongly influence perceptions of rivers ( Garcia *et al.*, 2020). Historical practices reinforce biases, but restoration can shift public attitudes. For example, a Dutch community surveyed near a planned restoration project reported not only greater feelings of safety but also improved scenic value, social connection, and recreational opportunities ( Verbrugge & van den Born (2018)). Engaging stakeholders in co-creation is central to fostering such change. Participatory approaches, emphasized by the EU Water Framework Directive, help integrate cultural values, strengthen legitimacy, and ensure success ( Polednikova & Galia, 2021; Schultz *et al.*, 2022).

### Policy integration

In Europe, riparian zones remain underrepresented in key policies such as the EU Water Framework Directive (WFD 2000/60/EC). Fragmented and sometimes conflicting frameworks—such as the WFD, Floods Directive, and Renewable Energy Directive—hinder integrated management ( Gumiero *et al.*, 2013). Defining riparian zones by fixed widths, for instance, overlooks their ecological complexity, limiting sustainable management (see CAP 23–27 https://agriculture.ec.europa.eu/common-agricultural-policy/cap-overview/cap-glance/key-policy-objectives-cap-2023-27_en ). A river basin approach based on “designing with nature” offers a more effective balance between ecological and social needs, reducing reliance on heavy-handed “river cleaning” ( Lilli *et al.*, 2020).

A coordinated policy framework is essential, integrating conservation, agriculture, and water management while recognizing riparian zones as part of broader ecosystems. In water-scarce regions, competition from agriculture and urban development places additional stress on these biodiversity hotspots ( Singh *et al.*, 2021; Urbanič *et al.*, 2022). Degradation of riparian vegetation and floodplains heightens flood risks and causes significant economic and ecological costs ( Hallegatte *et al.*, 2013; Poff *et al.*, 1997). Policymakers often underestimate these indirect costs, underscoring the need for broader recognition of riparian functions.

To protect both people and ecosystems, policy must move from reactive maintenance to proactive restoration, treating riparian zones as natural infrastructure for climate resilience ( Feld *et al.*, 2011; Wohl *et al.*, 2005). 

Initiatives such as the **COST Action CONVERGES** had highlight the value of synthesizing knowledge, improving stakeholder communication, and co-developing management tools ( https://converges.eu/converges/). To overcome entrenched biases and misinformation, scientists and policymakers should:

1. Communicate evidence transparently, countering myths with accessible explanations.
2. Engage communities through participatory processes that respect socio-cultural values.
3. Demonstrate benefits through visible examples of successful restoration.
4. Align policies across sectors to reduce contradictions and strengthen ecosystem-based management.

Adaptive, socio-ecological strategies emphasizing collaboration, stakeholder engagement, and ongoing monitoring are essential. Without addressing societal biases, decision-makers risk perpetuating outdated practices that degrade both ecosystems and resilience. Confronting climate and biodiversity crises requires moving from conflict toward co-creation, securing long-term benefits for both biodiversity and society.

## The role of citizen science in increasing science in riparian vegetation management

Citizen science plays a pivotal role in strengthening communication between researchers, policymakers, and the public. By engaging non-experts in data collection, it makes scientific information more accessible and socially relevant, while facilitating its integration into decision-making processes ( Bonney *et al.*, 2009; Hecker *et al.*, 2018). Such engagement also fosters trust in science, as individuals directly observe results in their own environments rather than perceiving scientific knowledge as distant or imposed ( Shirk *et al.*, 2012).

Data generated through citizen science should be curated in open-access repositories, validated by experts, and made available to all stakeholders ( European Commission, 2022). Importantly, participants should also be involved in interpreting results, combining local knowledge with scientific expertise to improve accuracy and legitimacy ( Danielsen *et al.*, 2005). Transparent sharing of outcomes with local authorities and communities ensures that results contribute to actionable policies and management strategies.

European initiatives such as OTTERS ( https://otters-eu.aua.am/), the Mission Ocean and Waters 2030, and the EU Citizen Science Platform are advancing standardized methodologies, allowing cross-country comparison and data aggregation at european scale. Such harmonization enhances scientific robustness, supports policymaking, and maximizes the impact of citizen science in addressing complex challenges such as riparian zone management, biodiversity loss, and climate adaptation ( Bonn *et al.*, 2016; Pocock *et al.*, 2019).

Participation further cultivates a sense of ownership and responsibility. When people actively contribute to monitoring and/or research, they are more likely to support conservation measures grounded in their own observations ( Dickinson *et al.*, 2012; Jordan *et al.*, 2012). For example, when local experiences—such as increased flooding following the removal of riparian vegetation—align with scientific predictions, communities become powerful advocates for evidence-based policies.

Well-designed citizen science projects for riparian zones should therefore aim to raise awareness about the ecological importance of riparian forests and their role in delivering essential ecosystem services ( Felitti *et al.*, 2020; Naiman & Décamps, 1997). Educational components are key to fostering deeper connections between people and rivers, while scientifically valid yet accessible monitoring protocols ensure inclusivity and reliability ( Kullenberg & Kasperowski, 2016).

### RiVe methodology

Although several large-scale initiatives exist to monitor water quality, hydrology, geomorphology, and biodiversity, no dedicated citizen science project has specifically focused on riparian forests—at least until now. To fill this gap, the RiVe methodology was developed in Italy, an innovative approach for monitoring riparian forest quality through the systematic collection of targeted data. It was created in 2020 in Italy by the National Biodiversity Network – ISPRA and the Citizen Science Observatory ( Gumiero *et al.*, 2024) ( https://www.nnb.isprambiente.it/vegetazioneriparia/inviasegnalazioni-it.html).

**Figure 3.**
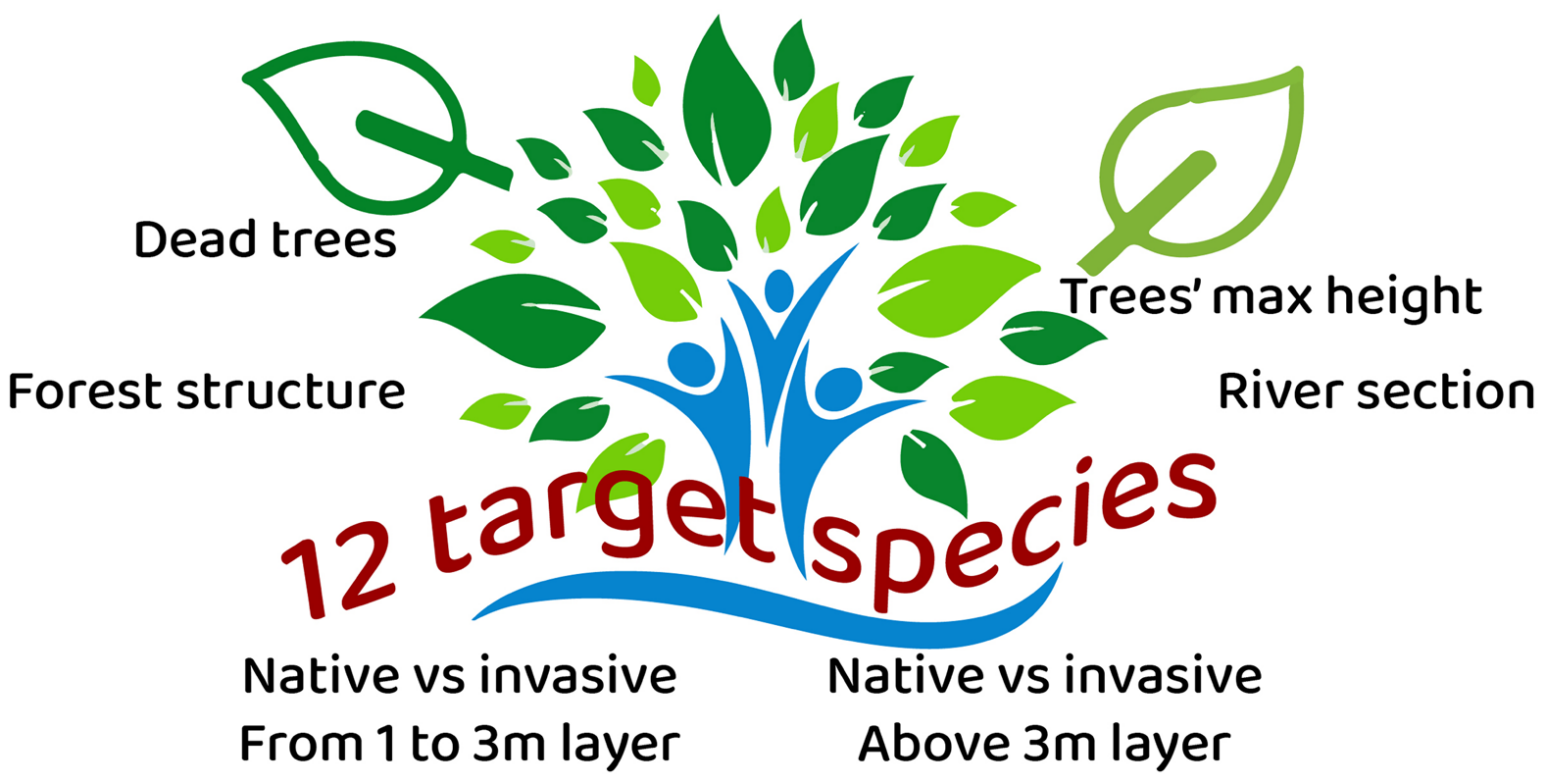
Questions asked in monitoring riparian forest with the RiVe method.

RiVe is structured around four key phases: volunteer training, data collection, data analysis, and dissemination of results to decision-makers and society ( Figure 3). Data collection is supported by an open-source smartphone app (ODK). During fieldwork, volunteers define a sampling area of approximately 10 × 15 meters and complete a form whose first four questions concern the river section, maximum height, presence of deadwood, and forest structure. Two additional questions address the presence and abundance of 12 target woody species across two layers (>3 m and 1–3 m). The 12 target species are selected by the local project expert following a common rationale: 4 softwood species (hygrophilous), 4 hardwood species (mesophilous), and 4 invasive species capable of reflecting the hydrological conditions and disturbance levels of the area. By diversifying the 12 target species in each local project according to the characteristics of the geographical area, the method can be applied on a European scale.The data collected are validated by project coordinators, ensuring scientific rigor. Finally, two indices can be calculated from the collected data: the Invasiveness Index (iRI) and the Riparian Forest Quality Index (iRiVe) (for mor details see the RiVe handbook https://otters-eu.aua.am/wp-content/uploads/2025/08/OTTERS_Monitoring_Riparian_Vegetation_through_Citizen_Science.pdf)

Moreover, the RiVe methodology can be integrated with other large-scale monitoring methods for riparian zones, such as remote sensing (see the QBR-GIS introduced by Segura-Méndez *et al.*, 2023).

## Conclusions – the way forward

The debate over riparian vegetation reflects a broader challenge: aligning scientific evidence, societal values, and political decision-making under increasing climate stress. For operational research, this is an opportunity to demonstrate the value of integrative, systems-based approaches that balance ecological, social, and economic dimensions.

Blaming riparian forests while ignoring the true drivers of flood risk — land-use change, urban encroachment, and insufficient restoration — will leave future generations with degraded rivers, heightened risks, and diminished ecosystem services. In contrast, embracing restoration offers the prospect of resilient rivers that enhance safety, biodiversity, and human well-being, often at lower long-term cost.

Operational research and policy integration can play a decisive role in bridging this divide by:

✓ **Multi-objective modeling** – weighing trade-offs between short-term interventions (e.g., dredging, cutting) and long-term resilience (restoration, floodplain reconnection).
✓ **Cost–benefit integration** – incorporating ecosystem services such as water purification, biodiversity, and carbon storage, often overlooked in traditional analyses.
✓ **Scenario analysis under climate change** – testing outcomes of “river cleaning” versus “room for rivers” approaches under extreme rainfall conditions.
✓ **Stakeholder engagement frameworks** – enabling participatory processes where communities, policymakers, and scientists co-design solutions, fostering legitimacy and trust.

The choice is clear:

✓ *Short-term reassurance* → “River cleaning” → High cost + low resilience.
✓ *Long-term resilience* → “Room for rivers” → Higher upfront investment + multiple co-benefits.

Within this context, the **OTTERS Project** highlights the vital role of citizen science in bridging communication gaps between researchers, policymakers, and the public. It advances scientifically robust, standardized methods to build a European network of comparable data and strengthens the role of participatory monitoring in decision-making. Central to this effort is the **RiVe method**, currently the only European approach dedicated to assessing riparian forest quality through a semi-quantitative framework. The method is being refined for continent-wide applicability and complemented by remote sensing, creating a powerful synergy between field-based and technological assessments.

Ultimately, citizen science is more than an educational tool: it is a driver of social change. By transforming passive observers into active participants, it reduces resistance to scientific recommendations, nurtures environmental stewardship, and contributes to more informed policies and sustainable river management.

## Disclaimer

*The views expressed in this article are those of the authors. Publication in OPEN RESEARCH EUROPE does not imply endorsement by the European Commission’s Horizon Europe funding programme*.

## Ethics and consent

Ethics and consent were not required.

## Data Availability

No data are associated with this article.
