# Supplementary material for: Citizen-engaged research for improved perceptions of riparian systems
Source: Open Res Eur. 2025 Oct 17;5:89. Originally published 2025 Mar 31. [Version 2] doi: 10.12688/openreseurope.19783.2 (PMC12663612; doi:10.12688/openreseurope.19783.2)
Supplement: Peer review history

# Reviewer report: Reviewer response for version 2

**DOI:** 10.21956/openreseurope.23293.r62994

**Published:** 2025-12-11

**Authors:** Ana Paula Portela

**Affiliations:** 1 Universidade de Lisboa Faculdade de Ciencias (Ringgold ID: 111161), Lisbon, Lisbon, Portugal

The authors present a citizen science methodology for riparian ecosystem monitoring developed in Italy which they argue can improve policies, decision-making and drive change in social perceptions about riparian vegetation.

 The topic is interesting; however, I believe the open letter would have benefited from a greater focus on the citizen science project being presented. Currently, the first sections of the letter are somewhat disconnected from the focus of the letter which is only presented in the last pages.

 The first key point to revise is the objective and use it to revise the remaining sections. I suggest focusing more on what seems to be the key contribution here – the citizens science monitoring project.

 There is also a missing link between the last paragraph of the introduction and the objective. I suggest adding some text explaining how citizen science can help overcome cultural beliefs and science denialism.

 The section on riparian ecosystem functions and services could be reduced and simultaneously improved. The definition of functions and services should be improved, and these should be clearly separated when presented. I also suggest removing or greatly reducing the bullet point description of ES. In my opinion this long description is taking attention away from the main point of letter.

 The section on social perceptions of riparian zones is interesting but currently lacks a more comprehensive view of contrasting views and adequate references in almost every paragraph. As mentioned in the text these views may vary greatly with socio-cultural context, therefore it is important to mention how and where biases exist. If possible, add references to studies, reports or news articles that can support your description of local views on riparian vegetation.

 In my opinion, the section on policy integration fails to describe how policies interact with the social views previously described.

 The section on citizen science is currently missing a good link with previous sections.  It is not immediately clear how citizen science relates with social beliefs and policies.

 The section on the Rive methodology seems to be the main novelty of the paper and thus should be expanded to present the methods in detail. For instance, it would be interesting to have an image of the app and a workflow of the field protocol.

 The conclusion section is mostly focused on topics that are not directly related to the citizen science project presented, thus should be revised to focus on the contributions of the project, what are the next steps, limitations, challenges and how others in the research community or otherwise can use this experience.

Where applicable, are recommendations and next steps explained clearly for others to follow? (Please consider whether others in the research community would be able to implement guidelines or recommendations and/or constructively engage in the debate)

Partly

Does the article adequately reference differing views and opinions?

Partly

Are all factual statements correct, and are statements and arguments made adequately supported by citations?

Partly

Is the rationale for the Open Letter provided in sufficient detail? (Please consider whether existing challenges in the field are outlined clearly and whether the purpose of the letter is explained)

No

Is the Open Letter written in accessible language? (Please consider whether all subject-specific terms, concepts and abbreviations are explained)

Yes

Reviewer Expertise:

Riparian ecology, ecosystem services

I confirm that I have read this submission and believe that I have an appropriate level of expertise to confirm that it is of an acceptable scientific standard, however I have significant reservations, as outlined above.

# Reviewer report: Reviewer response for version 2

**DOI:** 10.21956/openreseurope.23293.r62394

**Published:** 2025-11-28

**Authors:** Luca Gallitelli

**Affiliations:** 1 University of Roma Tre, Rome, Italy

Regarding the revised ms, the Authors addressed the comments of Reviewers and I think now it can be accepted for indexing. I only suggest checking English flow, figures, and the direct link to RiVe if it is working.

Where applicable, are recommendations and next steps explained clearly for others to follow? (Please consider whether others in the research community would be able to implement guidelines or recommendations and/or constructively engage in the debate)

Partly

Does the article adequately reference differing views and opinions?

Yes

Are all factual statements correct, and are statements and arguments made adequately supported by citations?

Yes

Is the rationale for the Open Letter provided in sufficient detail? (Please consider whether existing challenges in the field are outlined clearly and whether the purpose of the letter is explained)

Partly

Is the Open Letter written in accessible language? (Please consider whether all subject-specific terms, concepts and abbreviations are explained)

Yes

Reviewer Expertise:

riparian areas, freshwater ecology, plastic pollution

I confirm that I have read this submission and believe that I have an appropriate level of expertise to confirm that it is of an acceptable scientific standard.

# Reviewer report: Reviewer response for version 2

**DOI:** 10.21956/openreseurope.23293.r63001

**Published:** 2025-11-25

**Authors:** Goncalo Duarte, Sofia Santos

**Affiliations:** 1 Universidade de Lisboa, Lisbon, Lisbon, Portugal

This revision and report are the result of a review process that involved me and my colleague Sofia Santos.

 This is an interesting and well-written open letter that I believe focuses on an interesting topic, although in our opinion, it requires further revisions and details.

 Regarding the rationale and the key functions and services provided by the riparian vegetation, we believe that you need to support more adequately some of the statements about accusations against the environmental groups, and about the Emilia-Romagna example, when you claim the problem is more the land use than the riverbank vegetation. This way, you would have your statements supported by scientific outputs instead of having it look like an opinion.

 Regarding the recreational and cultural services, I believe you should expand your considerations to state that some studies concluded that engineered or managed landscapes have a higher aesthetic appeal. This seems to be a relevant caveat to consider here.

 You have used the term “biodiversity hotspots”, but we are unsure of what you mean.

 There is a specific definition of “ecological hotspot”, but this does not fit the context. Please verify this.

 The RiVe methodology is not sufficiently detailed; the authors need to highlight the advantages of this method, but also its limitations and shortcomings, which are absent from the text. A comparison with other existing methods is necessary so that readers can evaluate the RiVe methodology. This is also entangled with your statement that the method applies to the European scale, without mentioning any shortcomings that inevitably should occur, given the heterogeneity that exists across Europe.

 Specific comments:

 SECTION: Abstract

 “that provide flood regulation” – You might consider broadening the statement, since your study does not focus on these four specific functions. For example "provide many valuable functions and services, such as flood regulation.."

 “The Emilia-Romagna floods” – you might consider introducing the example more explicitly; "For instance, the debate about the underlying causes of the Emilia-Romagna floods (2023–2024) ilustrates..."

 “and climate intensification” – "climate change intensification"

 SECTION: Riparian forest: a fundamental component of watercourses

 “Riparian vegetation contributes coarse particulate organic matter (CPOM)” – It seems that this sentence is not right, maybe there is a word(s) missing between "contributes" and "coarse" (something like "to the").

 “Carbon Sequestration and ait quality improvement” – Not sure what “ait” is? could it be "air"?

 “Their aesthetic appeal enhances human well-being and cultural identity (Cameron et al., 2020; Thiele et al., 2019).” – Something to be considered for this point: There are studies indicating that sometimes natural riparian landscapes or riparian forests (those with higher ecological condition) have a lesser aesthetic appeal than "engineered" or managed riparian landscapes or forests. DOI: 10.1002/rra.3514

 SECTION: Societal perception of riparian zones and related biases

 “even accused of threatening public safety.” – Can you offer some reference, like a newspaper article, for instance? This is a relevant statement for activism and restoration planners; it would be good to have some support for it.

 “In Emilia-Romagna, levees, roads, and housing confined flows, making vegetation appear more hazardous than it is. Most large woody debris threatening infrastructure, like bridges, originates from upstream landslides or unstable banks, not intact riparian forests.” – I believe this requires a reference to a study made in this area or some very clear examples through pictures and maps from the area. Imagining someone wants to discredit this article, not having support here makes it easy to dismiss this relevant information.

 “Well-structured forests stabilize riverbanks and intercept debris before it reaches vulnerable sites.” – Please use a reference to support this sentence.

 “Yet research shows” – Provide several examples to have a more compelling argument.

 “Expanding floodplains, reconnecting rivers to natural corridors, and restoring space for water to spread are proven strategies to reduce flood peaks while preserving ecological integrity.” – Provide several examples to have a more compelling argument.

 “Expanding floodplains, reconnecting rivers to natural corridors, and restoring space for water to spread are proven strategies to reduce flood peaks while preserving ecological integrity. Selective interventions may be justified in urban contexts, but large-scale clearance undermines resilience. Even in dammed or channelized rivers, riparian trees can be partly maintained if the space between banks allows.” – The ecosystem service “flood regulation and mitigation” is correctly cited and well supported by the references provided, but they don't extend to these claims. Please, reinforce this section with references.

 “integrating conservation, agriculture, and water management” – Why are urban areas not included? Rivers in these areas are also relevant both ecologically and socially.

 “on these biodiversity hotspots” – What do you mean by biodiversity hotspots? The ecological definition of "hotspot" does not simply equate to place with high species richness (DOI: 10.1007/BF02240252). If in this case you just mean richness, then this would be the term to use.

 SECTION: The role of citizen science in increasing science in riparian vegetation management

 “such as increased flooding following the removal of riparian vegetation—align” – Caution with simplified examples. Given the complexity of flood dynamics and multiple factors involved, these relationships may not always be directly observable (within a given time frame). A straightforward cause-and-effect link could create unrealistic expectations.

 “RiVe methodology” – The authors need to highlight the advantages of this method but also it's limitations and shortcomings (which are absent). Also, how is this better than other existing methods? A comparison with other methods (using a table is a straightforward option) is necessary so that readers can evaluate the RiVe methodology.

 “(https://www.nnb.isprambiente.it/vegetazioneriparia/inviasegnalazioni-it.html)” – This link is not working.

 “(Figure 3)” – Figure 3 does not seem to be illustrative of this sentence.

 “(ODK)” – you might consider including the full name of the application (Open Data Kit – ODK) for readers who may not be familiar with the app.

 “Figure 3. Questions asked” – When the figure is mentioned in the text, it is not clear what it is illustrating.

 “complete a form whose first four questions concern the river section, maximum height, presence of deadwood, and forest structure.” – this is probably more related to what Figure 3 depicts.

 “By diversifying the 12 target species in each local project according to the characteristics of the geographical area, the method can be applied on a European scale.” – I think the authors should be more cautious about this statement. Riparian areas can be quite diverse across Europe. Reference species can be very different; using just 12 species could not be enough to assess ecological condition in a given area, and having hardly any species may not represent severe ecological issues in some areas.

 SECTION: Conclusions – the way forward

 “The method is being refined for continent-wide applicability” – This should be indicated before.

 “Ultimately, citizen science is more than an educational tool: it is a driver of social change. By transforming passive observers into active participants, it reduces resistance to scientific recommendations, nurtures environmental stewardship, and contributes to more informed policies and sustainable river management.” – The conclusion highlights very well the importance of citizen science as a driver of social change. However, it may also be worthing to note that riparian restoration alone cannot fully address extreme events, like Emilia-Romagna floods. In many places, especially urban areas, there is, for exemple, a structural lack of space that prevent rivers from functioning naturally, together with the growing unpredictability of extreme events. Even so, public involvement remains essential, for the reasons you state. I would simply suggest making this balance explicit, so that one dimension is not unintentionally softened in order to emphasise the other.

Where applicable, are recommendations and next steps explained clearly for others to follow? (Please consider whether others in the research community would be able to implement guidelines or recommendations and/or constructively engage in the debate)

Partly

Does the article adequately reference differing views and opinions?

Partly

Are all factual statements correct, and are statements and arguments made adequately supported by citations?

Partly

Is the rationale for the Open Letter provided in sufficient detail? (Please consider whether existing challenges in the field are outlined clearly and whether the purpose of the letter is explained)

Yes

Is the Open Letter written in accessible language? (Please consider whether all subject-specific terms, concepts and abbreviations are explained)

Yes

Reviewer Expertise:

Freshwater Ecology, ecological restoration, River Network functioning

We confirm that we have read this submission and believe that we have an appropriate level of expertise to confirm that it is of an acceptable scientific standard, however we have significant reservations, as outlined above.

# Reviewer report: Reviewer response for version 2

**DOI:** 10.21956/openreseurope.23293.r63000

**Published:** 2025-11-22

**Authors:** Yu Gong

**Affiliations:** 1 Wuhan Botanical Garden, Chinese Academy of Sciences, Beijing, Beijing, China

This study is benefical to understanding the riparian ecosystems, including their functions and services. However, several errors are needed to correct before publish.

 1. "ait quality" is incorrect. It is "air quality".

 2. what are "Blaming vegetation" and "Blaming riparian forests" means? Are you sure the word "blaming" is correct?

Where applicable, are recommendations and next steps explained clearly for others to follow? (Please consider whether others in the research community would be able to implement guidelines or recommendations and/or constructively engage in the debate)

Yes

Does the article adequately reference differing views and opinions?

Yes

Are all factual statements correct, and are statements and arguments made adequately supported by citations?

Yes

Is the rationale for the Open Letter provided in sufficient detail? (Please consider whether existing challenges in the field are outlined clearly and whether the purpose of the letter is explained)

Yes

Is the Open Letter written in accessible language? (Please consider whether all subject-specific terms, concepts and abbreviations are explained)

Yes

Reviewer Expertise:

My research is about  the carbon and nitrogen cycling in ripairan ecosystems.

I confirm that I have read this submission and believe that I have an appropriate level of expertise to confirm that it is of an acceptable scientific standard, however I have significant reservations, as outlined above.

# Reviewer report: Reviewer response for version 2

**DOI:** 10.21956/openreseurope.23293.r63003

**Published:** 2025-11-22

**Authors:** Lenka Kuglerová

**Affiliations:** 1 Swedish University of Agricultural Sciences, Umeå, Sweden

This open letter addresses an important and timely topic — the role of riparian vegetation in providing ecosystem services, the persistence of misconceptions about its management, and the potential of citizen science to bridge gaps between science, policy, and public perception. The subject matter is relevant in the context of European water and biodiversity policies, and the article is written in a clear, accessible style that would be understandable to readers across disciplines.

 The revised version shows substantial improvement in structure and flow compared to the earlier version. 

**Strengths**

- The paper synthesizes established ecological knowledge on riparian zones and presents it in a way that is accessible to non-specialist audiences.
- The discussion linking social perceptions, flood management narratives, and riparian ecology is well framed and relevant to current European debates.
- The inclusion of the RiVe methodology and its connection to citizen science provides a practical illustration of participatory approaches in environmental monitoring.
- References are mostly up to date and generally well chosen (but see my further comments).

**Limitations**

- The article offers limited novelty in terms of science or conceptual development. The ecological and policy arguments are well established and have been presented in numerous previous syntheses and reviews.
- The section on citizen science and the RiVe app remains descriptive rather than analytical and I see this as the main potential for improvement. There are no data or evaluation results demonstrating the method’s utility or effectiveness. It would be valuable if some results of the surveys could be presented, especially those that could support the arguments made here (that inclusion of citizen science can strengthen perceptions and policy decisions). For examples, I find figure 3 fairly uninformative (it only repeats what is written in the text) so replacing it by a table or figure presenting some of the RiVe outcomes seems like a low hanging fruit. 
- At times, the letter reads more as an advocacy piece than a scientific synthesis, which is acceptable for an open letter but limits its academic contribution.
- A few more citations are essential, especially on page 5, paragraphs starting with "The Emilia-Romagne floods of ...", and  "Blaming vegetation is politically...". I understand that this is an open letter but in both of these paragraphs are statements that need appropriate citations (e.g., "yet research shown that well managed riparian zones..." - no research that shows this is presented). 
- I also found some spelling issues and editorial suggestions: 
  - Page 3 last paragraphs "Flood regulation and mitigation. **C**omposed... (capital C). 
  - Page 4-5: When listing the ecosystem services and ecological functions, please be consistend with capital or lower case letter in their names. Sometimes capital letters are use (e.g., Shading and microclimate **R**egulation, Energy **I**nputs to food webs), while in other cases only the first letter is capitalized. 
  - Page 5: When you name the Emilia-Romagna flood, please specific country. The general audience will not know where it was. 
  - Figure 2: Please add where are those images taken from, by whom etc. 

In a traditional peer-reviewed research journal, the absence of new empirical results or conceptual advances would likely preclude publication. However, within the context of *Open Research Europe*, which emphasizes transparency, accessibility, and the sharing of perspectives rather than exclusivity, this open letter can be considered a useful contribution.

 It effectively communicates the need for better societal understanding of riparian systems and can demonstrates how citizen science initiatives can support that aim (if some more outcomes are added to support this claim).

 I therefore **recommend approval with reservations**, primarily due to the limited novelty and analytical depth, but recognizing the article’s communicative and policy relevance within the scope of this platform.

Where applicable, are recommendations and next steps explained clearly for others to follow? (Please consider whether others in the research community would be able to implement guidelines or recommendations and/or constructively engage in the debate)

Yes

Does the article adequately reference differing views and opinions?

Partly

Are all factual statements correct, and are statements and arguments made adequately supported by citations?

Partly

Is the rationale for the Open Letter provided in sufficient detail? (Please consider whether existing challenges in the field are outlined clearly and whether the purpose of the letter is explained)

Yes

Is the Open Letter written in accessible language? (Please consider whether all subject-specific terms, concepts and abbreviations are explained)

Yes

Reviewer Expertise:

Riparian and freshwater ecology, Riparian forest management, Biodiversity, Ecological functions.

I confirm that I have read this submission and believe that I have an appropriate level of expertise to confirm that it is of an acceptable scientific standard, however I have significant reservations, as outlined above.

# Reviewer report: Reviewer response for version 1

**DOI:** 10.21956/openreseurope.21401.r54702

**Published:** 2025-07-28

**Authors:** Olga Laiza Kupika

**Affiliations:** 1 University of Botswana, Gaborone, Botswana

The rationale for the paper isn't clear in terms of operational terms and the regional focus for the work. key terms should be clearly defined for clarity. I suggest the authors start by highlighting the policy basis for use of citizen science and how it is framed.  I addition, The article is rather generalised and does not specify the names of case study river systems/ basins to allow for additional reading.  Some Acronyms have not been adequately defined. The title is a bit misleading, while the article content focus on barriers and approaches, the misconceptions bit is not coming out clearly. General flow needs to be revised to ensure key aspects are connected.

Where applicable, are recommendations and next steps explained clearly for others to follow? (Please consider whether others in the research community would be able to implement guidelines or recommendations and/or constructively engage in the debate)

Partly

Does the article adequately reference differing views and opinions?

Partly

Are all factual statements correct, and are statements and arguments made adequately supported by citations?

Partly

Is the rationale for the Open Letter provided in sufficient detail? (Please consider whether existing challenges in the field are outlined clearly and whether the purpose of the letter is explained)

Partly

Is the Open Letter written in accessible language? (Please consider whether all subject-specific terms, concepts and abbreviations are explained)

Partly

Reviewer Expertise:

ecosystem services, local ecological knowledge climate change and biodiversity conservation

I confirm that I have read this submission and believe that I have an appropriate level of expertise to confirm that it is of an acceptable scientific standard, however I have significant reservations, as outlined above.

# Reviewer report: Reviewer response for version 1

**DOI:** 10.21956/openreseurope.21401.r53575

**Published:** 2025-07-04

**Authors:** Luca Gallitelli

**Affiliations:** 1 University of Roma Tre, Rome, Italy

The authors address the crucial topic of riparian habitat management. Given the relevance and timeliness of the subject, as well as the appropriate scope and content of the manuscript, the paper deserves to be indexed To ensure the highest quality, the manuscript would benefit from minor revisions and additions. Please consider my specific comments as suggestions to improve your manuscript.

1. Rationale and Aims:
  I recommend further elaborating on the study’s aims and hypotheses. Additionally, please clarify the specific objectives of your letter.
2. The concept of "riparian misconceptions" appears in both the title and the manuscript, but would benefit from a more detailed explanation in the text.
3. Two insightful papers that could enrich both the Introduction and Discussion sections are:
  - Refer to 3, 4
4. Consider including additional examples of work in riparian zones, such as: (Refer to 1, 2 )
5. If other examples of citizen science initiatives for monitoring riparian habitats exist, please include them. Additionally, it would be useful to compare traditional methods for sampling riparian habitats with the citizen science approach to highlight the strengths and potential of this technique. 
6. Regarding future management guidelines, although the RiVe example is well-discussed, the manuscript could be strengthened by providing more concrete guidelines for future riparian habitat management. How should these guidelines be implemented? Would they vary across different biogeographic zones?
7. Regarding the RiVe Protocol, to enhance its usefulness, consider adding more detailed steps to make it a replicable guideline for practitioners.
8. Have you considered using frameworks such as DPSIR (Drivers, Pressures, State, Impact, Response) or SWOT (Strengths, Weaknesses, Opportunities, Threats) for structuring your discussion on riparian habitat management? These may align well with your objectives.
9. Regarding the management of riparian zones, you may wish to expand your discussion on riparian habitat management. I would suggest adding insights from previous published research, (Refer to 5,6,7)
10. On the societal perception of Riparian Zones, I suggest merging the subparagraphs under this section into one or two coherent paragraphs for improved readability. This approach could also be considered for other sections.
11. The sentence “Riparian vegetation offers multiple ecosystem services (Figure 4):” would benefit from the inclusion of relevant references.
12. The manuscript could further emphasise the time scale involved in managing riparian zones. Additionally, please discuss any existing planned actions—at national or international levels—regarding practices such as mowing riparian vegetation along watercourses.
13. Figures: The figures are of good quality. Please ensure all captions and formatting conform to the journal’s style requirements.
14. Consider adding a dedicated paragraph outlining future directions and actionable recommendations based on your findings. Moreover, a paragraph on the limitations of your study and literature studies may be addressed.
15. Further details on the  EU Water Framework Directive and riparian health indices could be beneficial to understand the next directions related to river water quality.

Where applicable, are recommendations and next steps explained clearly for others to follow? (Please consider whether others in the research community would be able to implement guidelines or recommendations and/or constructively engage in the debate)

Partly

Does the article adequately reference differing views and opinions?

Yes

Are all factual statements correct, and are statements and arguments made adequately supported by citations?

Yes

Is the rationale for the Open Letter provided in sufficient detail? (Please consider whether existing challenges in the field are outlined clearly and whether the purpose of the letter is explained)

Partly

Is the Open Letter written in accessible language? (Please consider whether all subject-specific terms, concepts and abbreviations are explained)

Yes

Reviewer Expertise:

riparian areas, freshwater ecology, plastic pollution

I confirm that I have read this submission and believe that I have an appropriate level of expertise to confirm that it is of an acceptable scientific standard, however I have significant reservations, as outlined above.

## References

[1] : RIPARIANET - Prioritising riparian ecotones to sustain and connect multiple biodiversity and functional components in river networks. *Research Ideas and Outcomes* .2023;9: 10.3897/rio.9.e108807 10.3897/rio.9.e108807

[2] : Plastic Pirates sample litter at rivers in Germany – Riverside litter and litter sources estimated by schoolchildren. *Environmental Pollution* .2019;245: 10.1016/j.envpol.2018.11.025 545-557 10.1016/j.envpol.2018.11.025 30469125

[3] https://www.sciencedirect.com/science/article/pii/S0167880920300761.

[4] https://academic.oup.com/bioscience/article/70/6/501/5824857.

[5] https://www.mdpi.com/2073-4441/17/9/1358.

[6] https://www.sciencedirect.com/science/article/pii/S1462901124002211,.

[7] https://www.journals.uchicago.edu/doi/abs/10.1086/706104,.

# Response

**Published:** 2025-09-30

**Authors:** Bruna Gumiero

**Luca Gallitelli**, University of Roma Tre, Rome, Italy The authors address the crucial topic of riparian habitat management. Given the relevance and timeliness of the subject, as well as the appropriate scope and content of the manuscript, the paper deserves to be indexed To ensure the highest quality, the manuscript would benefit from minor revisions and additions. Please consider my specific comments as suggestions to improve your manuscript. For each comment see my replay in *italics*

1. Rationale and Aims:
  I recommend further elaborating on the study’s aims and hypotheses. Additionally, please clarify the specific objectives of your letter.

*You're right about the need to clarify the objectives, and I've actually revised and restructured the entire text, making the objectives clearer and more consistent with the rest of the test.*

1. The concept of "riparian misconceptions" appears in both the title and the manuscript but would benefit from a more detailed explanation in the text.

*I also clarified this concept well*

1. Two insightful papers that could enrich both the Introduction and Discussion sections are:

*Thanks for the tip but I thought I had discussed the ES aspect enough for an open letter anyway I added the two references in the bibliography*

1. Consider including additional examples of work in riparian zones, such as: (Refer to 1, 2 )

*Since this is already a long open letter, I think we should focus on the specific objectives.*

1. If other examples of citizen science initiatives for monitoring riparian habitats exist, please include them. Additionally, it would be useful to compare traditional methods for sampling riparian habitats with the citizen science approach to highlight the strengths and potential of this technique. 

*I developed this method because I didn't find anything similar in the literature, so as far as I know there are no others. Again, this is an open letter, but I've included the Braun-Blanquet method used, slightly modified. I've also included a link to the English manual, which clearly explains everything.*

1. Regarding future management guidelines, although the RiVe example is well-discussed, the manuscript could be strengthened by providing more concrete guidelines for future riparian habitat management. How should these guidelines be implemented? Would they vary across different biogeographic zones?

*I have added the link to the Rive manual where you can find all the information relating to RiVe. And yes the methodology takes into account the different geographical areas, giving the possibility of choosing the 12 target species for each specific project *

1. Regarding the RiVe Protocol, to enhance its usefulness, consider adding more detailed steps to make it a replicable guideline for practitioners.

*I have added the link to the Rive manual where you can find all the information relating to RiVe*

1. Have you considered using frameworks such as DPSIR (Drivers, Pressures, State, Impact, Response) or SWOT (Strengths, Weaknesses, Opportunities, Threats) for structuring your discussion on riparian habitat management? These may align well with your objectives.

*Thanks for the suggestion but the goal was already ambitious in itself for CS activities, furthermore the two indexes could certainly be useful if one wants to use them within the DPSIR*

1. Regarding the management of riparian zones, you may wish to expand your discussion on riparian habitat management. I would suggest adding insights from previous published research, (Refer to 5,6,7)

*As mentioned above, although it is one of the objectives of the RiVe method: that is, to provide useful information for the sustainable management of riparian vegetation, management is not the focus of this open letter, so it has been rightly mentioned given its importance but not explored in depth.*  

1. On the societal perception of Riparian Zones, I suggest merging the subparagraphs under this section into one or two coherent paragraphs for improved readability. This approach could also be considered for other sections.

*Having sorted out the whole text I hope I have managed to answer this one*

1. The sentence “Riparian vegetation offers multiple ecosystem services (Figure 4):” would benefit from the inclusion of relevant references.

*the references are already in the text and in any case I have added two further suggested references*

1. The manuscript could further emphasise the time scale involved in managing riparian zones. Additionally, please discuss any existing planned actions—at national or international levels—regarding practices such as mowing riparian vegetation along watercourses.

*This is an open letter focused on riparian forest monitoring, which could be a useful tool for managing these areas. However, management is not one of the objectives, so I didn't consider it worth going into further detail to avoid making it too long.*

1. Figures: The figures are of good quality. Please ensure all captions and formatting conform to the journal’s style requirements.

done

1. Consider adding a dedicated paragraph outlining future directions and actionable recommendations based on your findings. Moreover, a paragraph on the limitations of your study and literature studies may be addressed.

*I think the new revised text has answered this comment*

1. Further details on the  EU Water Framework Directive and riparian health indices could be beneficial to understand the next directions related to river water quality.

*The WFD, while acknowledging the importance of riparian vegetation, has not currently provided for any monitoring of riparian vegetation; it is only superficially mentioned in some applications within the geomorphological index. Furthermore, the Hydrogeomorphological index in Italy is currently used only in elevated stretches to confirm them. Therefore, riparian vegetation is currently not directly involved in the definition of the Good Ecological Status of rivers.*
